# DNA damage response inhibitors enhance tumour treating fields (TTFields) potency in glioma stem-like cells

**DOI:** 10.1038/s41416-023-02454-0

**Published:** 2023-09-30

**Authors:** Aurelie Vanderlinden, Callum G. Jones, Katie N. Myers, Ola Rominiyi, Spencer J. Collis

**Affiliations:** 1 Division of Clinical Medicine, The University of Sheffield, School of Medicine and Population Health, Sheffield, S10 2RX UK; 2 Division of Neuroscience, The University of Sheffield, School of Medicine and Population Health, Sheffield, S10 2RX UK; 3 Department of Neurosurgery, Royal Hallamshire Hospital, Sheffield Teaching Hospitals NHS Foundation Trust, Sheffield, S10 2JF UK

**Keywords:** Targeted therapies, DNA damage response

## Abstract

**Background:**

High-grade gliomas are primary brain cancers with unacceptably low and persistent survival rates of 10–16 months for WHO grade 4 gliomas over the last 40 years, despite surgical resection and DNA-damaging chemo-radiotherapy. More recently, tumour-treating fields therapy (TTFields) has demonstrated modest survival benefit and been clinically approved in several countries. TTFields is thought to mediate anti-cancer activity by primarily disrupting mitosis. However, recent data suggest that TTFields may also attenuate DNA damage repair and replication fork dynamics, providing a potential platform for therapeutic combinations incorporating standard-of-care treatments and targeted DNA damage response inhibitors (DDRi).

**Methods:**

We have used patient-derived, typically resistant, glioma stem-like cells (GSCs) in combination with the previously validated preclinical Inovitro™ TTFields system together with a number of therapeutic DDRi.

**Results:**

We show that TTFields robustly activates PARP- and ATR-mediated DNA repair (including PARylation and CHK1 phosphorylation, respectively), whilst combining TTFields with PARP1 or ATR inhibitor treatment leads to significantly reduced clonogenic survival. The potency of each of these strategies is further enhanced by radiation treatment, leading to increased amounts of DNA damage with profound delay in DNA damage resolution.

**Conclusion:**

To our knowledge, our findings represent the first report of TTFields applied with clinically approved or in-trial DDRi in GSC models and provides a basis for translational studies toward multimodal DDRi/TTFields-based therapeutic strategies for patients with these currently incurable tumours.

## Introduction

Brain tumours kill more children and adults under 40 than any other cancer, with the high-grade gliomas (glioblastoma WHO grade 4) being the most common tumours arising within the brain and, contributing to around 190,000 deaths/year globally [1, 2]. The current standard-of-care therapy for these incurable tumours is de-bulking surgical resection followed by a therapeutic regimen of combined radio- and chemotherapy, utilising the DNA alkylating agent Temozolomide (TMZ) alongside ionising radiation, followed by cycles of TMZ alone [3, 4]. Around half of glioblastomas exhibit promoter hypermethylation, leading to reduced levels of the dealkylating enzyme MGMT that specifically and directly removes alkylation lesions induced by TMZ and is, therefore, an established biomarker of TMZ effectiveness and short-term clinical response [5]. However, despite this, favourable MGMT promoter methylation status is unfortunately not sufficient to confer acceptable long-term survival, with 5-year survival rates even for patients with tumours with methylated MGMT promotor regions is only around 14% [4]. In addition to large amounts of inter- and intra-tumoural genetic and transcriptomic heterogeneity [6–18], one of the main reasons ascribed to the high levels of treatment resistance and near universal recurrence exhibited by glioblastomas is the presence of difficult-to-treat glioma stem-like cell (GSC) subpopulations [19, 20], which possess unlimited regenerative potential and exhibit enhanced DNA repair pathway activity [21–26]. As such, median time to disease recurrence is only around 7 months, resulting in median survival rates around 10–16 months and 5-year overall survival rates less than 10% for patients diagnosed with a glioblastoma, which unfortunately have improved very little over the last 40–50 years [6, 27, 28].

Tumour treating fields (TTFields) therapy is a non-invasive therapy which delivers low-intensity (1–3 V/cm) intermediate-frequency (100–500 kHz) alternating electric fields to localised tumour sites [29]. Importantly, TTFields therapy (200 kHz) represents the first clinically-approved treatment for glioblastoma in over 10 years [29], supported by the extension of overall survival by ~5 months in patients with newly diagnosed glioblastoma within a landmark randomised clinical trial led by Stupp and colleagues [30]. Molecular evidence suggests that TTFields, through the exertion of physical forces on polar macromolecules, can cause a plethora of biological effects within cells [29] including: interference with mitotic spindle assembly, enhancement of cell membrane and blood-brain barrier permeability, effects on cancer cell motility, induction of immunogenic cell death, induction of replication stress and impairment of DNA damage response mechanisms (DDR) [31–41]. Since DNA-damaging chemoradiotherapy is the standard-of-care for post-surgical glioblastoma management, the discovery of TTFields modulatory effects on DNA damage and highly-coordinated cellular DNA damage response (DDR) processes [38–40, 42] presents a critical opportunity to develop more effective, rationally-designed TTFields-based therapeutic strategies [29, 43, 44]. Therefore, using clinically-relevant GSC models [24, 25], we investigated combining established therapeutic and preclinical DDR inhibitors (DDRi [44, 45]) with TTFields and radiation as part of important preclinical evaluation studies to determine if such strategies could be developed clinically to enhance TTFields potency in the treatment of currently incurable glioblastoma.

## Materials and methods

### Cell culture

G1 and G7 patient-derived primary GSCs were kindly gifted by Professor Colin Watts (University of Birmingham, Brain Cancer Programme Chair) and Professor Anthony Chalmers (University of Glasgow, Chair of Clinical Oncology), which were initially derived from freshly resected anonymised glioblastoma specimens by Professor Watts’ former laboratory in Cambridge [24, 25, 46]. All GSCs were propagated as adherent monolayers on matrigel-coated T75 flasks. Cells were grown in advanced DMEM supplemented with L-glutamine (Invitrogen, 25030081), B27 (Invitrogen, 17504-044), N2 (Invitrogen, 17502-048), Penicillin-Streptomycin (Invitrogen, 15140122), Heparin (Sigma, H3393-10KU), amphotericin B (Gibco, 15290), EGF (100 μg/ml, Invitrogen, PHG0313) and FGF (100 μg/ml, Invitrogen, PHG0263), in a humidified incubator at: 37 °C, 5% CO_2_ and 21% O_2_.

### Inhibitor and irradiation treatments

ATR inhibitor AZD6738 (Selleckchem; S7693; ATRi) and PARP1 inhibitor Olaparib/Lynparza/AZD2281 (Adooq Bioscience; A10111; PARPi) were diluted with DMSO to make 10 mM stocks and were stored at −20 °C. A dose range was used to determine the optimal effective and non-toxic dose of each inhibitor (Supplementary Fig. S1A, B). Cells were treated with the DDRi at the indicated concentrations or with vehicle control only (DMSO). DMSO and all DDRi were diluted in stem media to the final intended concentrations and 2 mL of the drug/DMSO dilutions was added to the desired wells. DMSO at concentrations equivalent to the drug solutions (<2%) were used as the vehicle control in all experiments. One hour following DDRi treatment, cells were then treated with either ionising radiation (IR) or sham irradiated. Cells were irradiated in a Caesium-137 (^137^Cs) Irradiator (CIS IBL437c) to a total dose of either 2 Gy or 5 Gy IR, as indicated in the figures. In all experiments, unirradiated control plates were taken out of the incubator for the same duration as the treatment plates to minimise experimental variation and act as a “sham” radiation control such that control plates were subjected to comparable environmental changes as experienced by cells during the irradiation process. After irradiation/sham, the cells were then exposed to TTFields for 48 h.

### TTFields treatments

The inovitro^TM^ system (NovoCure Ltd; Haifa, Israel) was used to generate TTFields [47]. G1 and G7 GSCs were seeded onto sterile, matrigel-coated glass coverslips in 12-well plates at a density of 3–5 × 10^4^ cells/well. Following seeding, cells were incubated overnight to allow for cell adhesion to the coverslips. The following day, the matrigel-coated coverslips with attached cells were transferred into inovitro^TM^ ceramic dishes (one coverslip/dish), which contained two pairs of electrodes positioned orthogonally for the delivery of TTFields, and 2 thermistors for measuring the temperature inside the dish. Dishes to receive TTFields treatment were connected to a generator to produce alternating electric fields at the frequency clinically-approved for the treatment of glioblastoma (200 kHz) with the directionality of electric fields treatment applied alternating by 90^o^ every 1 s [47]. As the delivery of the electric fields generates micro heating within the dish, dependant on the intensity of the applied field, the base plate with connected dishes was placed in a refrigerated incubator (with 5% CO2 and 21% O2) in order to maintain the temperature of the treated dishes at 37 °C throughout the treatment. The incubator was set at a temperature of 22 °C equating to a maintained intensity of 1.33 V/cm RMS at 37 °C [47]. Cells were treated for a duration of 48 h based on calculated cell doubling times as determined by prior cell growth assays (Supplementary Fig. S1C).

### Clonogenic survival assays

Following treatment with DDRi, IR and/or TTFields, cells were harvested and re-seeded in matrigel-coated 6-well tissue culture plates at varying densities (300 and 500 cells/well) (specified in the results). Cells were incubated for 21 days, then stained with methylene blue, and the resulting colonies (cluster of 50 cells or more) were counted. The plating efficiency (PE) was determined for untreated control populations (colonies counted/cell plated) and the surviving fraction (SF) for each experimental condition was calculated relative to the untreated control; number of counted colonies/ (number of cells plated x PE).

### Western blotting

Between 25–50 μg of protein and 4x NuPage LDS Loading Buffer mix were loaded into each lane of a NuPAGE 4–12% Bis-Tris gradient gel and electrophoresed for ~75 min at 150 V. Proteins were then transferred to nitrocellulose membranes at 100 V for 120 min in Mini PROTEAN Tetra Cells, using 1x NuPAGE transfer buffer (20X stock) diluted with pure methanol and ddH_2_O. Membranes were blocked for 60 min in 5% milk with phosphate-buffered saline (Thermo Fisher Scientific, BR0014) with 5% Tween-20 (Sigma, P1379) (PBS-T) or 5% bovine serum albumin (BSA) (Sigma, A2153) with TBS-T, when blotting for pChk1 Ser345. Membranes were incubated with primary antibodies overnight at 4 °C with anti β-actin (Santa Cruz, sc-47778; 1:5000), anti-pChk1 Ser345 (Cell signalling, #2341; 1:500), anti-Chk1 (Cell signalling, #2360; 1:1000), anti-ATR (R&D Systems, #AF4717; 1:250), anti-PARP1 (Santa Cruz, sc-8007; 1:1000), anti-αPAR (Millipore, MABE1016; 1:1000), or anti-γH2AX Ser139 (Santa Cruz, sc517348; 1:1000). Primary antibodies were made up in 5% milk with PBS-T or 3% BSA with TBS-T, again when blotting for pChk1. Membranes were washed 3x with PBS-T, each wash lasting 10 min. Membranes were then incubated with secondary antibodies conjugated to HRP all at 1:1000 in 5% milk with PBS-T for 1 h: anti-rabbit (DAKO, P0399), anti-goat (DAKO, P0449) or anti-mouse (DAKO, P0447). Membranes were washed 3 times in PBS-T and protein bands visualised using Pierce ECL western blotting substrate and developed using medical x-ray film and a Konica SRX 101 A Processor.

### Immunofluorescence

Cells were seeded onto sterile Matrigel-coated coverslips in 12-well plates at a density of 3 × 10^4^ cells/well. At the end of TTF/IR/DDRi treatment, cells were fixed with 4% Paraformaldehyde (PFA; Santa Cruz Biotechnology, SC-281692) for ten minutes and subsequently washed twice with PBS. Cells were permeabilised with 0.5% Triton X-100 (Thermo Fisher Scientific, A16046) in PBS for 10 min. Once permeabilised, cells were washed three times with PBS and blocked for 1 h with 3% BSA in PBS. Cells were then incubated overnight at 4 °C with primary antibodies; phospho-histone ser139 (γH2AX) antibody (Millipore, JBW301; 1:500) and p53-binding protein 1 (53BP1) antibody (Abcam, ab36823; 1:500) in 1% BSA PBS. Following incubation with primary antibodies, cells were washed three times with PBS. Cells were then incubated with the secondary antibodies Alexa Fluor 488-conjugated anti-rabbit antibody (Life Technologies, A-11034; 1:500) and Alexa Fluor 555-conjugated anti-mouse antibody (Life Technologies, A11005; 1:500) made up in PBS with 1% BSA at room temperature for 1-h in the dark (wrapped in foil). Finally, coverslips were washed three times in PBS, including a final wash in PBS containing 2 μg/ml DAPI before being mounted onto microscope slides using Shandon Immu-Mount medium (Thermo Fisher Scientific, 9990402). Slides were left to dry overnight at room temperature in the dark. Microscopy was performed on a Nikon Eclipse T200 inverted microscope (Melville), using a 100x objective lens. Individual 53BP1 foci in each cell nucleus were counted, and/or cells were scored as either positive (≥5 foci) or negative (<5 foci) for γH2AX or pRPA2 (T21) staining. A minimum of 100 cells were analysed for each experimental condition per slide.

### Comet assays

The comet assay kit (Trevigen; 4250-050-K) was used to process samples. At the end of treatment, cells were collected and resuspended in warm PBS, and then pelleted at ~180rcf for 3 min and washed twice with warm PBS. Cell pellets were then resuspended in 1 mL warm PBS and cells were counted using a haemocytometer. A final cell suspension of 1 × 10^5^ cells/mL in PBS for each sample was produced. 12.5 μL of cell suspension was then mixed with 112.5 μl LMagarose (4250-050-02; 1:10 dilution) and 100 μLof the LMagarose/cell mix was pipetted onto the sample area of a comet slide (Trevigen; 4250-050-03). Cells were stored flat at 4 °C in the dark for 30 min to promote adherence of the suspension, then lysed with COMET Lysis Solution (4250-050-01) at 4 °C for 30 min in the dark. Following lysis, cells were exposed to Alkaline Unwinding Buffer (200 mM NaOH (Sigma; S5881), 1 mM EDTA (Trevigen; 4250-050-04) for 20 mins at room temperature. Electrophoresis was carried out at ~21 V, with a constant current of 300 mA (achieved by adjusting the volume of Alkaline Electrophoresis Buffer (200 mM NaOH, 1 mM EDTA (Sigma; 1233508)) for 30 mins at 4 °C. Slides were rinsed twice with H_2_O and were then immersed in 70% ethanol for 5 min. Samples were dried overnight at room temperature. To stain cells, 100 µl 10,000X SYBR Gold Solution (Invitrogen; S11494) made up at 1:30,000 in TE buffer (10 mM Tris-HCl, Sigma; 10812846001) pH 7.5, 1 mM EDTA (Sigma; 1233508) was pipetted onto each sample area and left to stain for 30 min in the dark. Excess SYBR Gold solution was removed by gently tapping the slides and dipping them in H_2_O. Slides were allowed to dry before imaging. A least 50 cells per condition were imaged using the FITC channel and 20x lens on a Nikon Eclipse TE200 Fluorescent Microscope. Images were analysed using TriTek COMET Score software (AMSBiotechnology, 2010) to determine the tail moment, which was used as a direct measure of DNA damage.

### Flow cytometry

The Biosciences kit (#556547) was used to process samples as described in the manufacturer’s protocol. At various time points following treatment (as specified in the results), media from each dish was collected and transferred to a labelled centrifuge tube. Cells were lifted and washed twice with cold PBS and resuspended in 100 μL 1X Binding Buffer (10X Binding Buffer; 0.1 M HEPES, pH 7.4; 1.4 M NaCl; 25 mM CaCl_2_; diluted to 1X in ddH_2_O, Biosciences, 556454). 5 μL Annexin V (27 μg/mL, Biosciences; 556419) and 5 μL Propidium Iodide (PI, Biosciences; 556463) were added to each sample and cells were incubated at room temp for 15 min in the dark. A further 200μL Binding Buffer was added to each tube and the cell suspension was then transferred into labelled FACS tube and analysed by BD LSR II Flow Cytometer. 10,000 cells/sample were counted on the LSRII and the resulting data was analysed using FlowJo software.

### Statistical analyses

Statistical significance was calculated using the nonparametric Mann–Whitney *U*-test comparing the indicated treatment to DMSO controls or to another indicated treatment cell population, and represented as follows: ns = not significant, *p < 0.05, **p < 0.01, ***p < 0.001, and ****p < 0.0001.

## Results

### PARPi enhances TTFields-mediated cell death in GSCs

**Fig. 1:**
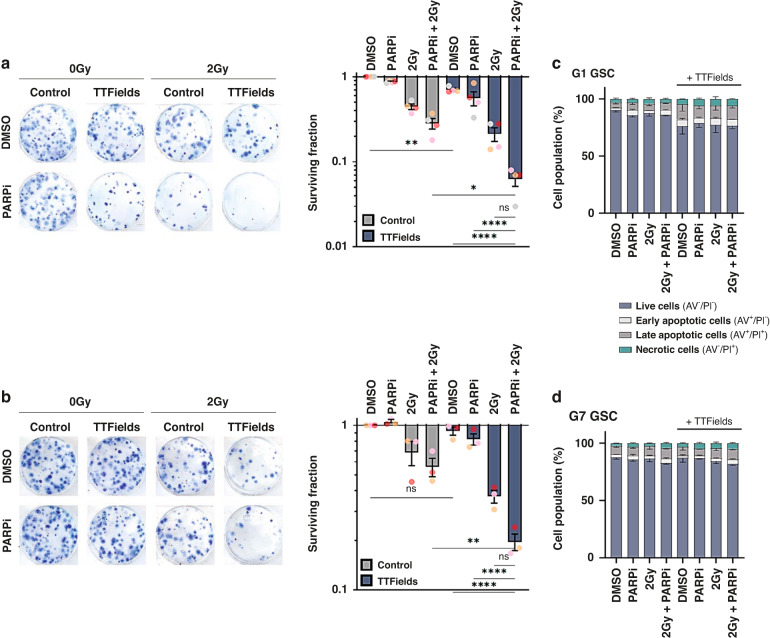
Olaparib potentiates TTFields cytotoxicity in glioma stem cells. **a** Left panel; representative images of colony formation of G1 GSC treated with PARPi, IR and/or TTFields as indicated. Right panel; clonogenic survival of G1 GSCs treated as indicated. **b** Same as in A:, but for G7 GSCs. **c** Measurements of apoptosis and necrosis in G1 GSCs treated as indicated. **d** Same as in **c**, but for G7 GSCs. Data shown represents the means derived from at least three independent biological repeat experiments along with their respective standard errors. Bars above the data highlight statistical significance calculations between the two indicated cell populations.

TTFields have previously been shown to elicit DNA damage and replication stress in human cancer cells and interfere with the efficient repair of radiation-induced DNA lesions [38–40, 42]. Furthermore, recent work using established lung cancer cell lines has also shown that co-application of TTFields with radiation and/or PARP1 inhibitors (PARPi) impart enhanced cell killing effects [39], and PARPi have been shown to successfully cross the blood-brain barrier (BBB) to enable therapeutically effective doses at glioma tumour sites in patients [48]. Consistent with previous findings in established cell lines, exposure of primary glioma stem-like cells (GSCs) to therapeutically relevant frequency of TTFields (200 kHz) caused DNA damage and activated both PARP1 and ATR signalling pathways (Supplementary Fig. S1D). Given this, and the current therapeutic intertest in PARPi for gliomas [48], we therefore assessed if combining PARPi with or without additional therapeutically relevant ionising radiation (IR) doses in primary GSCs could augment TTFields potency. Indeed, combination of PARP1 inhibition with the therapeutic compound Olaparib (Lynparza™) in the G1 GSC model augmented TTFields potency, which was further and dramatically enhanced when combined with 2 Gy IR (Fig. 1a). Importantly, a similar enhanced cytotoxic effect on clonogenic survival was also independently observed in the G7 GSC model (Fig. 1b). Importantly, these effects were not due to large amounts of early post-treatment apoptosis induced prior to plating for the 3-week clonogenic assays (Fig. 1c, d), suggesting that aberration of long-term DNA repair capacity might be a potential mechanism (see later). It is also interesting to note that decreased clonogenic survival was more pronounced in G1 GSCs compared with G7 GSCs, which could be a consequence of a greater inherent IR and PARPi resistance, possibly linked to the enhanced basal PARP1 activity in the G7 GSCs compared with G1 GSCs (Supplementary Fig. S1E).

### TTFields-PARPi combinations yield elevated and prolonged DNA damage in GSCs

**Fig. 2:**
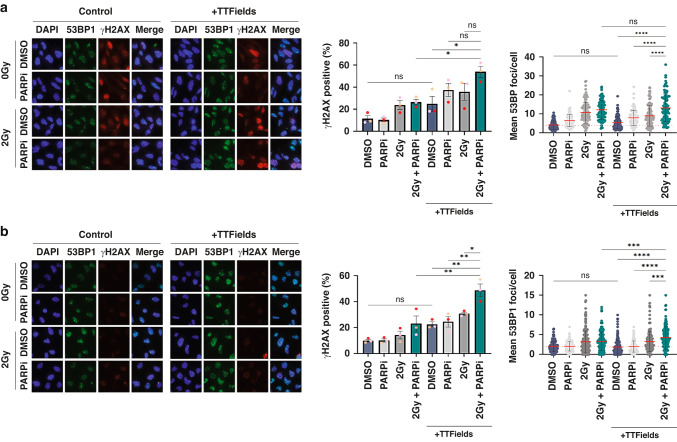
PARPi elevates DNA damage levels induced by TTFields-IR combinations. **a** Left panel; Representative immunofluorescent images of the indicated G1 GSC population stained for either 53BP1 (green; DSB marker) or γH2AX (red; general DNA damage marker) after the indicated treatment combinations. DAPI DNA stain (blue) was used to identify cell nuclei for scoring purposes. Right panel; quantification of γH2AX positive cells (%) or mean 53BP foci/nucleus in the indicated G1 GSC cell populations. **b** Same as in **a**, but for G7 GSCs. Red dashed line indicates the mean in DMSO only treated population. Data shown on the graphs represents the either means derived from at least three independent biological repeat experiments along with their respective standard errors or collated data from at least three independent biological repeat experiments along with their respective standard deviations. Bars above the data highlight statistical significance calculations between the two indicated cell populations.

**Fig. 3:**
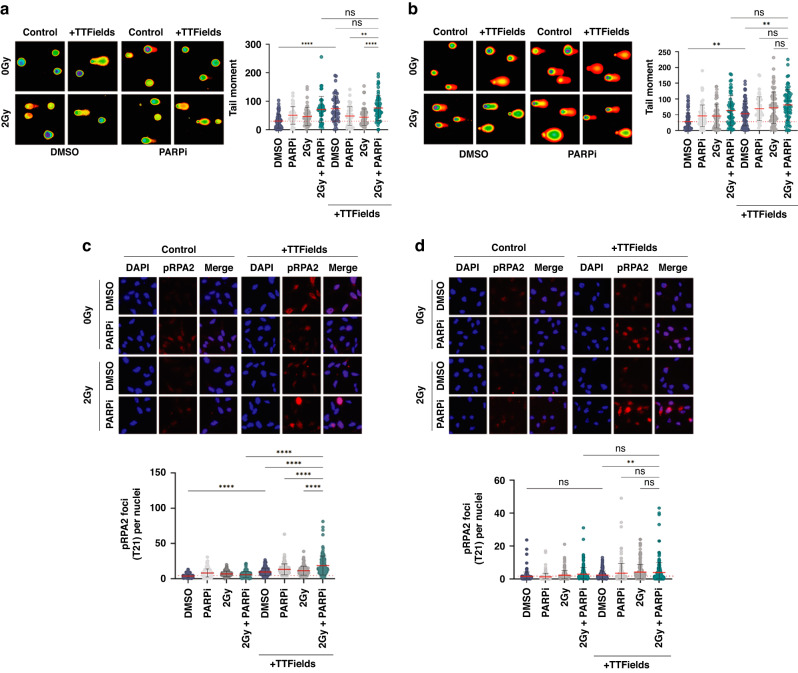
Further assessment of DNA damage and replication stress induced by combining PARPi with TTFields and IR treatments. **a** Representative Comet images of the indicated G1 GSC population with quantification of tail moment from at least three independent biological repeat experiments shown to the right. **b** Same as in A:, but for G7 GSCs. **c** Upper panel; Representative immunofluorescence images of the indicated G1 GSC cell population stained for pRPA2 (red) and DAPI (blue) used to identify nuclei. Lower panel; quantification of pRPA2 positive cells in the indicated G1 GSC populations. **d** Same as in **c**, but for G7 GSCs. Red dashed line indicates the mean in DMSO treated population. Data shown on the graphs represents collated data derived from at least three independent biological repeat experiments along with their respective standard deviations. Bars above the data highlight statistical significance calculations between the two indicated cell populations.

Consistent with the clonogenic survival data, we determined that both G1 and G7 GSCs treated with PARPi-TTFields combinations exhibited elevated levels of DNA damage that was further and dramatically enhanced when combined with 2 Gy IR (Fig. 2a, b, respectively). Furthermore, direct assessment of DNA damage by Comet assays confirmed enhanced levels of DNA damage in both G1 and G7 populations treated with PARP1-TTFields and PARPi-IR-TTFields combinations compared with those treated with either modality alone (Fig. 3a, b). In keeping with the differential clonogenic survival and apoptotic index between combination treated G1 and G7 GSCs (Fig. 1), the increased levels of DNA damage were more elevated in G1 GSCs compared with G7 GSCs (Figs. 2, 3), which is consistent with the inter-tumoural heterogeneity and inherent treatment sensitivity/resistances to DNA damaging agents commonly observed within and across different gliomas [44].

Given that TTFields have been shown to enhance replication stress by downregulation of FA/BRCA repair pathway genes, and PARP1 is important for DNA replication fork stabilisation during replication stress [39], we assessed the effects of the combination treatments on replication stress through quantifying the presence of phosphorylated RPA2 which plays a key role in stabilising single-stranded DNA at stalled replication forks and recruiting downstream DDR mediators [49]. PARPi, IR and TTFields all individually led to modestly increased replication stress, which was again more enhanced in G1 compared to G7 GSC (Fig. 3c, d). Additionally, the various combinations of PARPi, IR and TTFields led to further significant increases in replication stress in G1 but not in G7 GSCs (Fig. 3c, d). Collectively, these data suggest that although some of the DNA damage induced by the combination treatments may be a consequence of elevated replication stress leading to fork collapse, other mechanisms may be involved in the enhanced cytotoxicity (reduced clonogenic survival) conferred by these combinations.

**Fig. 4:**
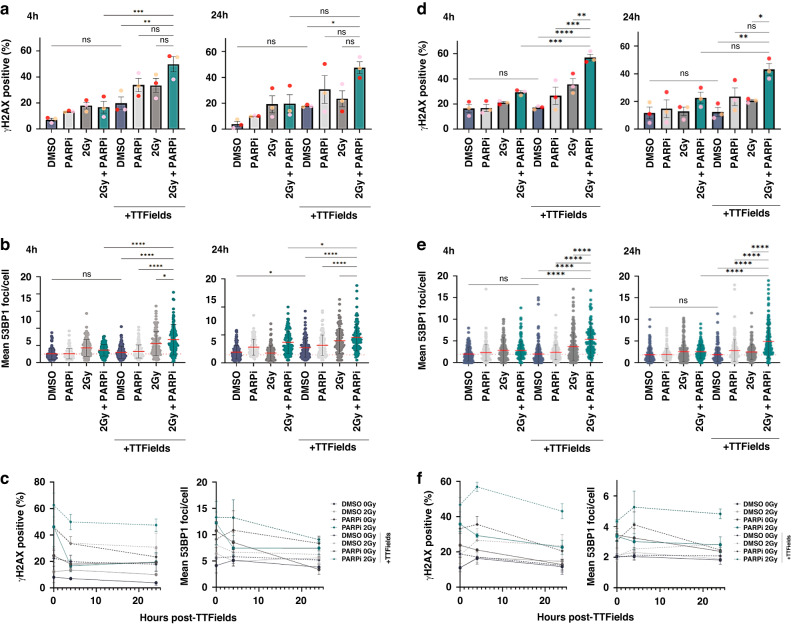
PARPi impedes the efficient resolution of TTFields-IR induced DNA damage. **a**, **b** Respective quantification of γH2AX positive cells (%) or mean 53BP foci/nucleus in the indicated G1 GSC cell populations at 4 h and 24 h post-treatment (see Supplementary Fig. S2A). Note: the 0 h timepoint data isn’t shown for space purposes and is a further three independent repeats of the data shown in Fig. 2 but it is shown on the line graph below. **c** Line graphs showing the data above together with the 0 h time point data for this set of experiments for the indicated DNA damage marker and treated G1 GSC population. **d**, **e** same as for **a**–**c**, but for G7 GSCs. Note: representative 4 h and 24 h images for γH2AX and 53BP are shown in Supplementary Fig. S2B. Red dashed line indicates the mean in DMSO treated population. Data shown on the graphs represents either means derived from at least three independent biological repeat experiments along with their respective standard errors or collated data from at least three independent biological repeat experiments along with their respective standard deviations with calculated statistical significance values shown as outlined in the material and methods section. Bars above the data highlight statistical significance calculations between the two indicated cell populations.

Therefore, in order to investigate the prolonged effects on DNA damage induced by the various combination treatments, we assessed the levels of DNA damage at both early (4 h) and late timepoints (24 h) following completion of 48 h of treatment with PARPi, IR and/or TTFields, as described above, using immunofluorescent quantification of the respective general DNA damage and double-strand break markers γH2AX and 53BP1. Similar to that observed immediately following TTFields dosing (Fig. 2), PARPi-IR-TTFields combination treated G1 GSCs exhibited significantly higher levels of DNA damage (~2-fold) compared with either PAPP1i-TTFields or IR-TTFields treatments, with elevated levels of DNA damage still remaining 24 h post-treatment (Fig. 4a–c and Supplementary Fig. S2A). Although overall, less DNA damage was induced in G7 GSCs compared with G1 GSCs (Fig. 2) comparable effects on long-term DNA damage were observed in G7 GSCs (Fig. 4d–f and Supplementary Fig. S2B).

### Preclinical assessment of ATRi in combination with IR and TTFields in GSCs

**Fig. 5:**
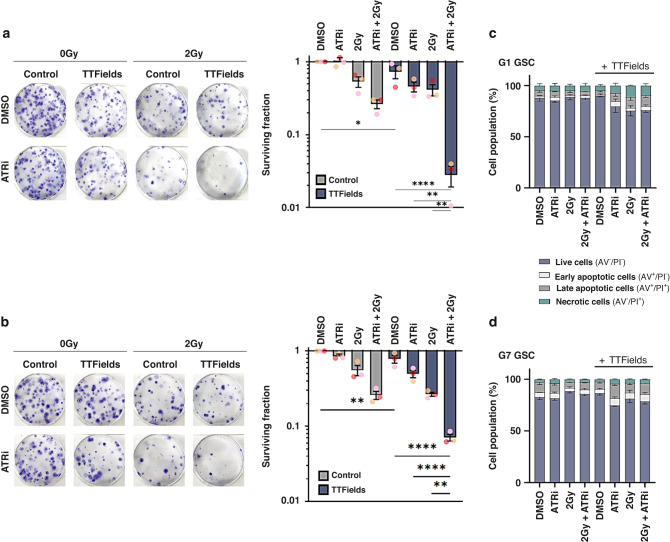
The ATRi AZD6738 potentiates TTFields cytotoxicity in glioma stem cells. **a** Left panel; representative images of colony formation of G1 GSC treated with ATRi, IR and/or TTFields as indicated. Right panel; clonogenic survival of G1 GSCs treated as indicated. **b** Same as in A:, but for G7 GSCs. **c** Measurements of apoptosis and necrosis in G1 GSCs treated as indicated. **d** Same as in **c**, but for G7 GSCs. Data shown represents the means derived from at least three independent biological repeat experiments along with their respective standard errors. Bars above the data highlight statistical significance calculations between the two indicated cell populations.

**Fig. 6:**
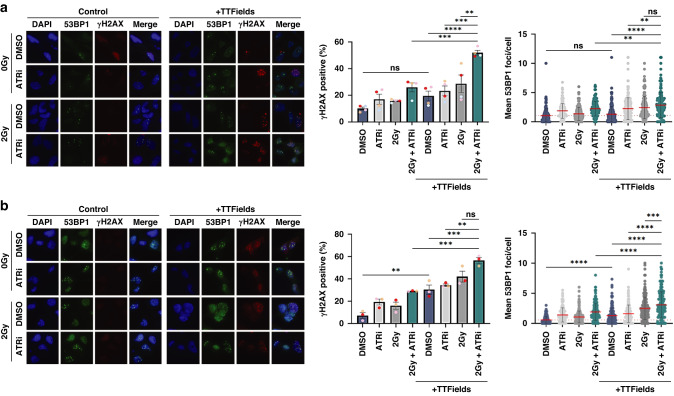
ATRi elevates DNA damage levels induced by TTFields-IR combinations. **a** Left panel; Representative immunofluorescent images of the indicated G1 GSC population stained for either 53BP1 (green; DSB marker) or γH2AX (red; general DNA damage marker) after the indicated treatment combinations. DAPI DNA stain (blue) was used to identify cell nuclei for scoring purposes. Right panel; quantification of γH2AX positive cells (%) or mean 53BP foci/nucleus in the indicated G1 GSC cell populations. **b** Same as in A:, but for G7 GSCs. Red dashed line indicates the mean in DMSO treated population. Data shown on the graphs represents the either means derived from at least three independent biological repeat experiments along with their respective standard errors or collated data from at least three independent biological repeat experiments along with their respective standard deviations. Bars above the data highlight statistical significance calculations between the two indicated cell populations.

**Fig. 7:**
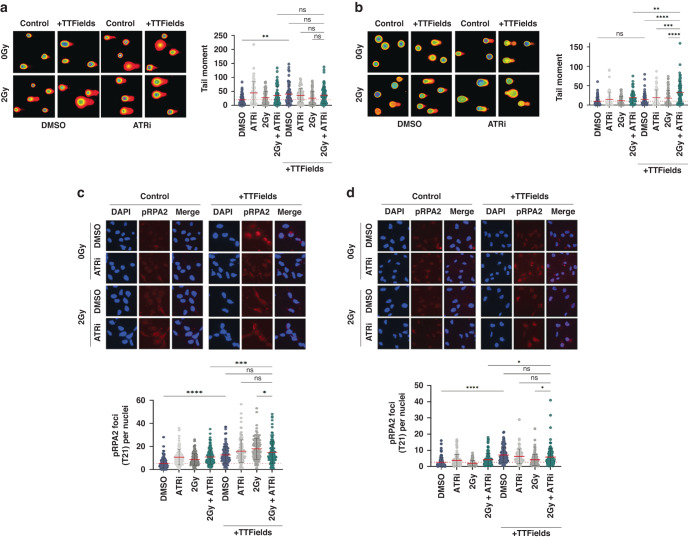
Further assessment of DNA damage and replication stress induced by combining ATRi with TTFields and IR treatments. **a** Representative Comet images of the indicated G1 GSC population with quantification of tail moment from at least three independent biological repeat experiments shown to the right. **b** Same as in A:, but for G7 GSCs. **c** Upper panel; Representative immunofluorescence images of the indicated G1 GSC cell population stained for pRPA2 (red) and DAPI (blue) used to identify nuclei. Lower panel; quantification of pRPA2 positive cells in the indicated G1 GSC populations. **d** Same as in **c**, but for G7 GSCs. Red dashed line indicates the mean in DMSO treated population. Data shown on the graphs represents collated data derived from at least three independent biological repeat experiments along with their respective standard deviations. Bars above the data highlight statistical significance calculations between the two indicated cell populations.

Given the key role of the ATR-mediated signalling pathway in cellular response to DNA damage and replication stress, the ATR kinase is an established oncology drug target in a range of tumours, including gliomas [44, 45]. For these studies, we focused on the use of the BBB-penetrant ATR inhibitor AZD6738 (ATRi), which is currently being assessed in several clinical trials, including in combination with the PARP1i Olaparib, and also in combination with radiotherapy [44, 45, 50]. We found that combinations of relatively non-toxic doses of ATRi with IR and TTFields led to significantly reduced clonogenic survival in both G1 and G7 GSCs (even more pronounced than the reduced surviving fractions observed with PARPi), which was also not associated with significantly enhanced early apoptotic processes (Fig. 5). As with the PARPi combinations, the ATRi-IR-TTFields combination was particularly potent in G1 GSCs compared with G7 GSCs (Fig. 5). The increased cytotoxicity in both GSC models was accompanied by elevated levels of DNA damage as assessed using the immunofluorescent markers γH2AX and 53BP1 (Fig. 6). Unexpectedly however, this only correlated with a significant increase in DNA damage as measured by Comet assay in G7 GSCs (Fig. 7a, b), and neither G1 or G7 combination-treated cell populations exhibited elevated levels of replication stress as measured using pRPA2 foci formation (Fig. 7c, d). This is particular intriguing given that both TTFields alone and ATRi treatment prior to IR enhanced radiation-induced pRPA2 foci formation in both G1 and G7 GSCs (Fig. 7c, d), which suggests that this may simply reflect stalled forks being converted into DNA breaks which releases RPA from ssDNA. Additionally, previous work from others has shown that GSCs have a greater capacity to repair DNA damage after ATRi-IR combination treatments than their bulk (non-stem) counterparts [24].

**Fig. 8:**
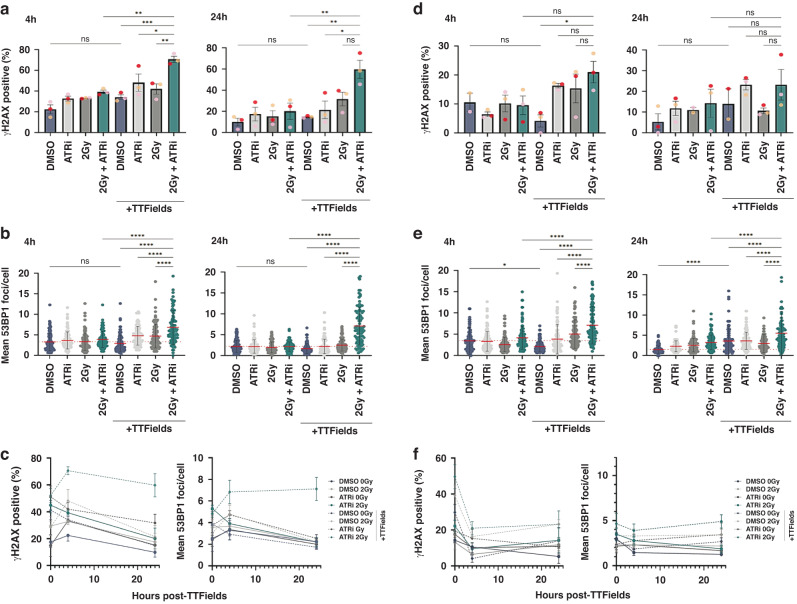
ATRi impedes the efficient resolution of TTFields-IR induced DNA damage. **a**, **b** Respective quantification of γH2AX positive cells (%) or mean 53BP foci/nucleus in the indicated G1 GSC cell populations at 4 h and 24 h post-treatment (see Supplementary Fig. S3A). Note: the 0 h timepoint data isn’t shown for space purposes and is a further three independent repeats of the data shown in Fig. 6 but it is shown on the line graph below. **c** Line graphs showing the data above together with the 0 h time point data for this set of experiments for the indicated DNA damage marker and treated G1 GSC population. **d**, **e** same as for **a**–**c**, but for G7 GSCs. Note: representative 4 h and 24 h images for γH2AX and 53BP are shown in Supplementary Fig. S3B. Red dashed line indicates the mean in DMSO treated population. Data shown on the graphs represents the either means derived from at least three independent biological repeat experiments along with their respective standard errors or collated data from at least three independent biological repeat experiments along with their respective standard deviations with calculated statistical significance values shown as outlined in the material and methods section. Bars above the data highlight statistical significance calculations between the two indicated cell populations.

Therefore, in order to assess to DNA damage levels in more detail, we carried out time-courses analyses of G1 and G7 GSCs following treatment with either ATRi, IR or TTFields alone, or in various combinations as described above. Akin to the results for PARPi (Fig. 4), pre-treatment of both G1 and G7 GSCs with ATRi prior to combination IR-TTFields treatment led to elevated and persistent levels of DNA damage (Fig. 8 and Supplementary Fig. S3). Although consistent with our other findings, peak and delayed resolution of DNA damage was substantially more pronounced in G1 GSCs compared with G7 GSCs (Fig. 8a–c and 8D–F, respectively), which exhibited nearly 4-fold increased DNA damage over basal levels even at 24 h post-treatment (Fig. 8c). However, even in the inherently more resistant G7 GSCs, combining ATRi with IR and TTFields led to significantly elevated levels of DNA damage and DNA breaks 24 h post-treatment, whereas the single agent or dual combination treated cells had returned to basal levels of DNA damage (Fig. 8f). Collectively, these data together with our data for PARPi in these GSC models, highlight the potential for DDRi combinations to enhance the efficacy and potency of TTFields therapeutics in the treatment of high-grade gliomas.

## Discussion

To our knowledge, we present here the first report of combining TTFields with DNA repair inhibitors in glioma stem cells and the first reported use of combining ATRi with TTFields. Importantly, our data are consistent with recent work by others showing the effectiveness of combining TTFields with PARPi and IR in non–small-cell lung cancer cell lines [39]. The profoundly increased sensitivity to TTFields that we observe in primary GSCs by pre-treatment with either PARPi or ATRi is particular exciting given that both PARPi and ATRi have been shown to exhibit good safety profiles in human trials and are able to reach the glioma tumour site through penetrating the blood-brain/tumour barrier. As such, both are currently being assessed in a range of glioma-focused clinical trials as part of both monotherapy approaches and in combination with current standard-of-care TMZ or IR therapies [48, 50–56]. This is particularly interesting given that TTFields have previously been shown to enhance BBB permeability [41], which could further improve the effective dose of such compounds at the tumour site. With regards to this, it is interesting and important to note that in addition to the use of clinically relevant 2 Gy IR doses throughout our study, the 0.5 μM dose of Olaparib that we used in this study is within the range of drug concentrations observed at murine orthotopic tumour sites and equivalent to the median clinically available concentration detected within the tumour core and resection margins in human tissue from a recent clinical trial [48]. The clinical evaluation of various DDRi to treat glioblastoma represents a rapidly developing area globally, including concerted efforts in the UK to establish a national adaptive early phase interventions platform to generate high-quality pharmacokinetic/pharmacodynamic (PK/PD) data on targeted therapy and multi-modal combinations from surgically resected tumour tissue. Our data, therefore, provides further important preclinical evaluation of the potential to combine these compounds as well as other DDRi with current standard-of-care therapies for gliomas [44], including TTFields, which has been clinically approved in numerous countries for both newly-diagnosed and recurrent gliomas [29].

Collectively, to help inform and prioritise candidate strategies for assessment within the rapidly evolving clinical trial landscape, our data suggest that PARPi and ATRi, as well as other DDRi in combination with IR, TMZ and TTFields, are worth investigating within a larger panel of primary, patient-derived GSC models representing a range of molecular and phenotypic contexts to further build on data generated in these studies using G1 and G7 GSCs which both represent MGMT methylated glioblastoma (IDH wildtype).

A current limitation to our current work is that these data have been generated in 2D GSCs that are several passages away from their primary tumour resection [24, 25], and have been shown to be amenable to 3D culture that yield more clinically-relevant drug responses [46, 57]. The main reason for this is that although there are preclinical TTFields devices available and in development for in vivo studies [29, 58], presently, no defined protocols for the delivery of TTFields in such 3D culture models are available [58]. However, very recent work has started to explore the possibility of delivering effective doses of TTFields within ex vivo 3D glioma models [59], and we have also recently been able to develop effective and robust delivery of TTFields within 3D GSC cultures (unpublished data). As such, based on the findings presented here, we are now carrying out subsequent evaluation of PARPi, ATRi and other DDRi in combination with TTFields within primary ex vivo 3D GSC models.

Other aspects worth considering when taking our findings presented here forward into further preclinical models is the often “left behind” post-surgical residual disease and the inherent inter- and intra-tumour heterogeneity that exists within these tumours, and how these traits can impact responses to radio-chemotherapy treatments and overall patient survival [6, 10]. In order to address this, we have developed an ongoing living biobank of over 110 GSC models derived from over 55 individual patients that have undergone surgical resection of their gliomas, which incorporates multiple models that recapitulate both intra-tumoural heterogeneity (multi-region sampling) and typically post-surgical residual disease using adjacent, invaded brain within *en-bloc* partial lobectomy specimens (Rominiyi et al., in revision). We therefore plan to also harness these models together with our recently developed 3D GSC TTFields protocols to provide further preclinical evaluation of PARPi, ATRi and other DDRi combinations to augment the efficacy of TTFields alone and in combination with current standard-of-care TMZ and IR therapies, and to also assess the potential pan-tumour efficacy of such approaches.

### Supplementary information

Supplementary Figure legends

Supplementary Figure S1

Supplementary Figure S2

Supplementary Figure S3

## Data Availability

The data that support the findings of these studies are available on request from the corresponding authors.
