# Supplementary material for: DNA damage response inhibitors enhance tumour treating fields (TTFields) potency in glioma stem-like cells
Source: Br J Cancer. 2023 Sep 30;129(11):1829–40. doi: 10.1038/s41416-023-02454-0 (PMC10667536; doi:10.1038/s41416-023-02454-0)
Supplement: Supplementary file 1 — Supplementary Figure legends [file 41416_2023_2454_MOESM1_ESM.docx]

**Supplementary Figure S1.**

**A:** Western blots showing effective PARP inhibition (reduction in PARylation) in G1 and G7 GSCs with the indicated doses of Olaparib. **B:** Same as in A:, but for ATRi using reduction in IR-induced phosphorylation of the ATR substrate CHK1 on Ser345 to assess inhibition of ATR kinase activity. **C:** Growth curves for G1 and G7 stem cells. The cell doubling time was calculated from the growth phase of the curves as detailed in the materials and methods section. G7 stem cells were calculated to have a doubling time of 1.6 days and G1 stem cells of 1.3 days. **D:** and **E:** Western blots of protein extracts from GSCs probed for the indicated proteins.

**Supplementary Figure S2.**

**A:** and **B:** representative immunofluorescence images of G1 and G7 GSC stained respectively for γH2AX or 53BP1 at the indicated time post singular or PARPi-IR-TTFields combination treatment. The collated data associated with these images from three independent repeat experiments are shown in Figure 4.

**Supplementary Figure S3.**

**A:** and **B:** representative immunofluorescence images of G1 and G7 GSC stained respectively for γH2AX or 53BP1 at the indicated time post singular or ATRi-IR-TTFields combination treatment. The collated data associated with these images from three independent repeat experiments are shown in Figure 8.
