# Supplementary figures and images for: DNA damage response inhibitors enhance tumour treating fields (TTFields) potency in glioma stem-like cells
Source: Br J Cancer. 2023 Sep 30;129(11):1829–40. doi: 10.1038/s41416-023-02454-0 (PMC10667536; doi:10.1038/s41416-023-02454-0)

**A****G1 GSC**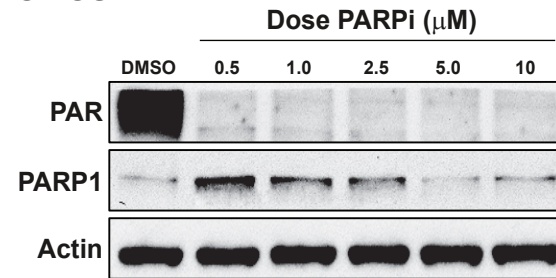**G7 GSC**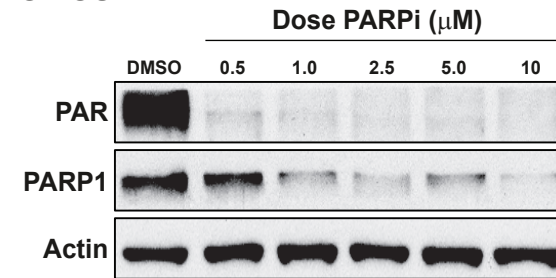**B****G1 GSC**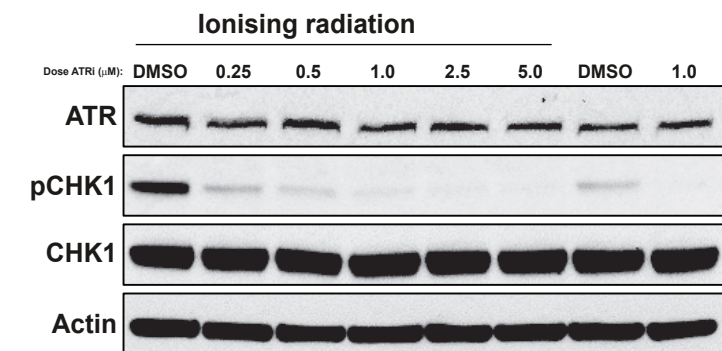**G7 GSC**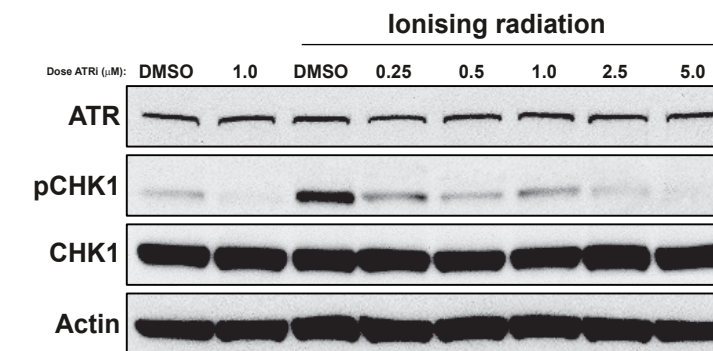**C**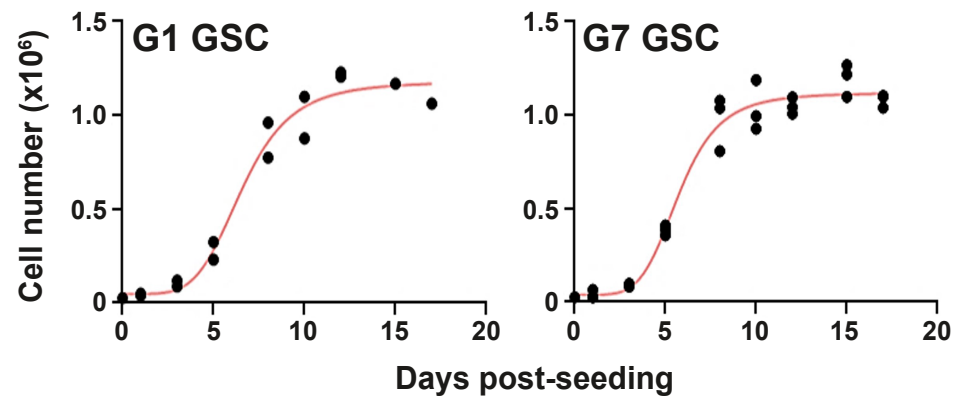**D**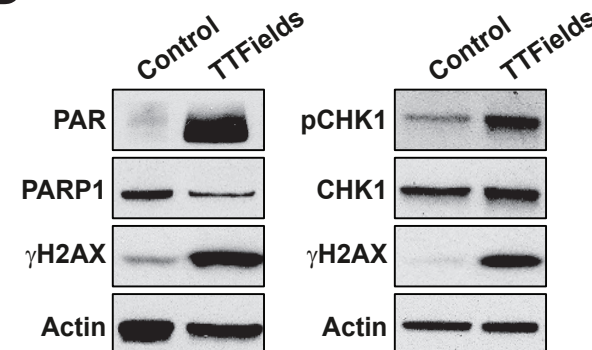**E**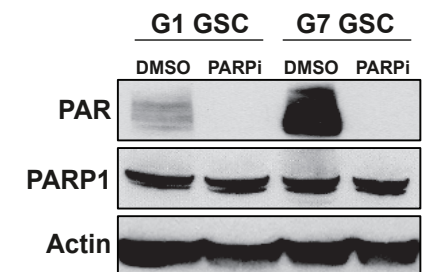

Supplement: Supplementary file 2 — Supplementary Figure S1 [file 41416_2023_2454_MOESM2_ESM.pdf]

**A**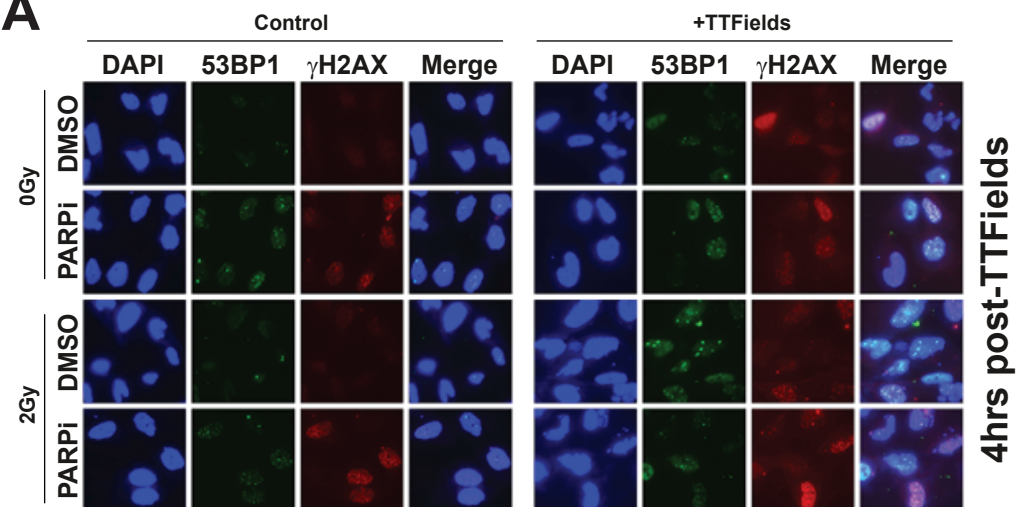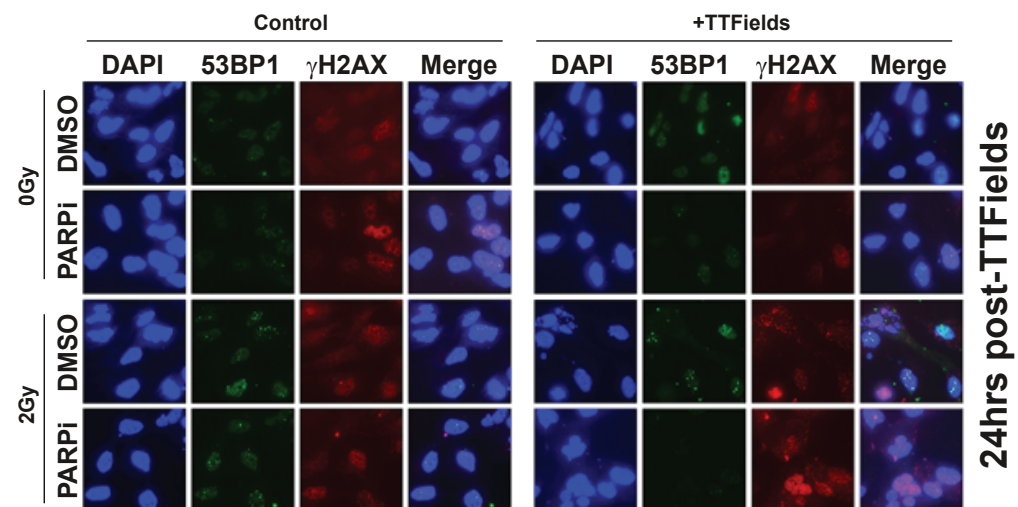**B**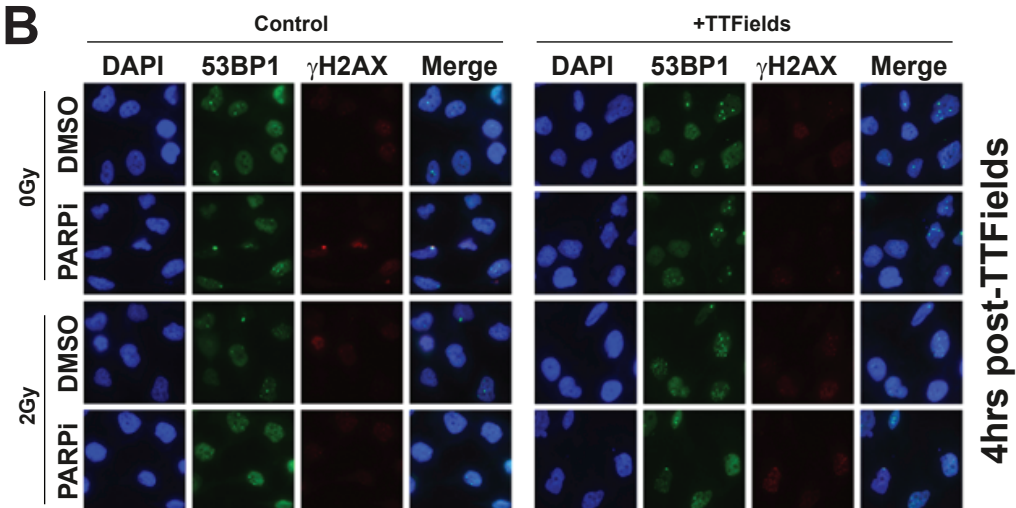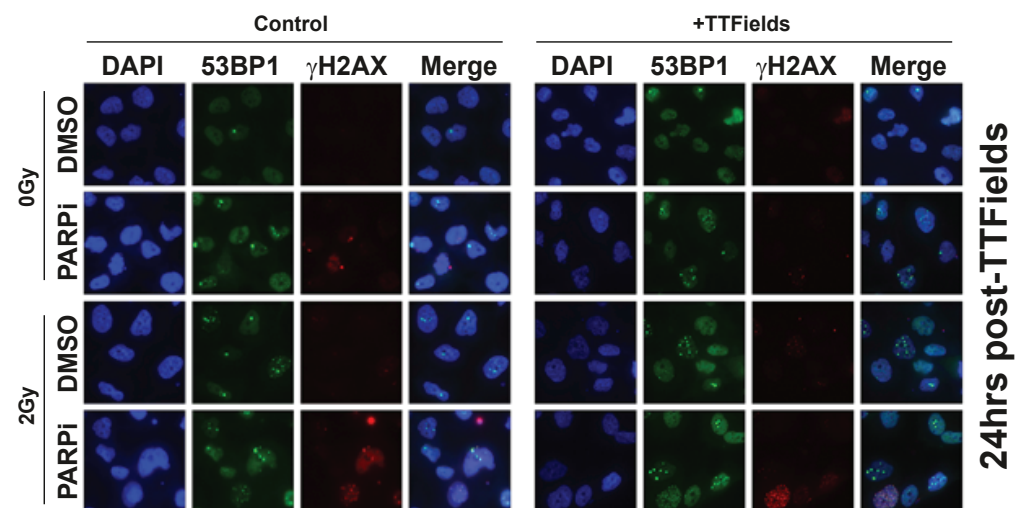

Supplement: Supplementary file 3 — Supplementary Figure S2 [file 41416_2023_2454_MOESM3_ESM.pdf]

**A**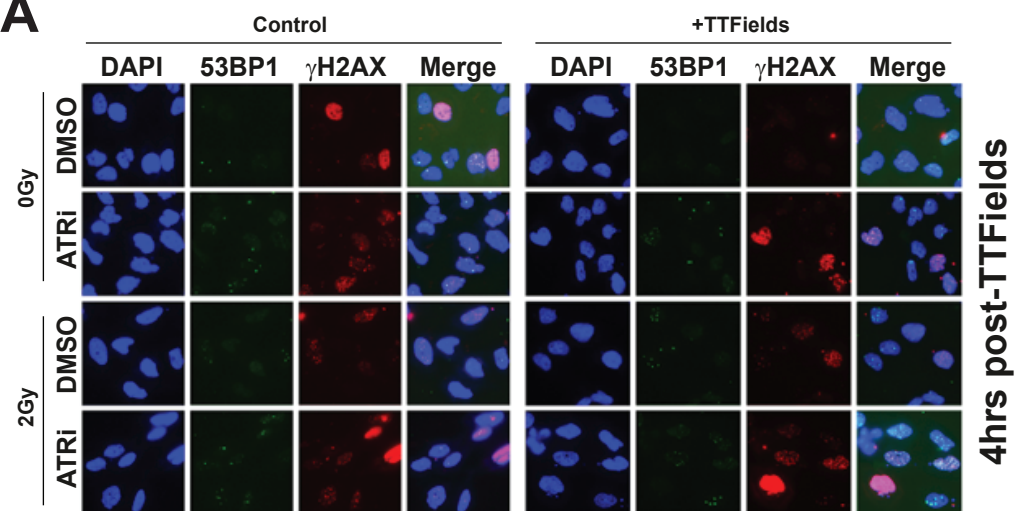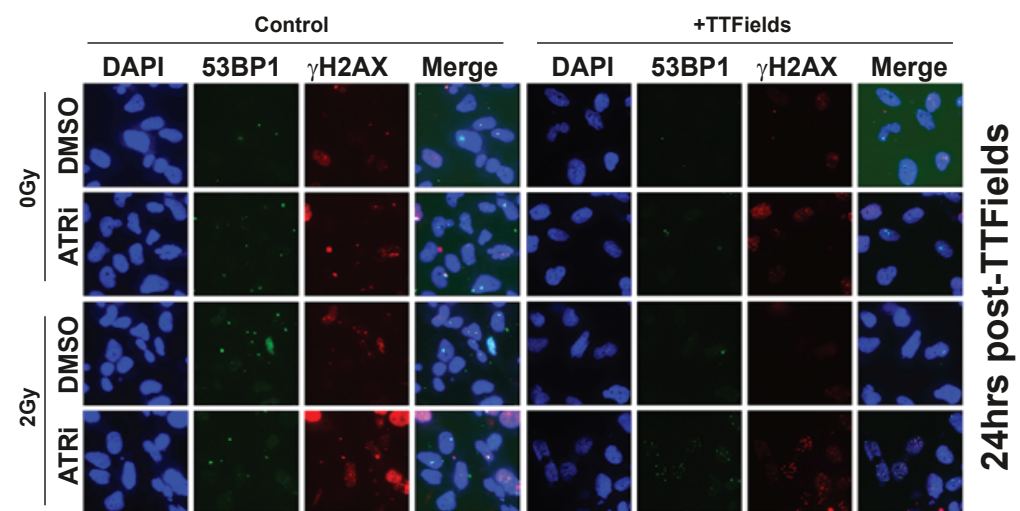**B**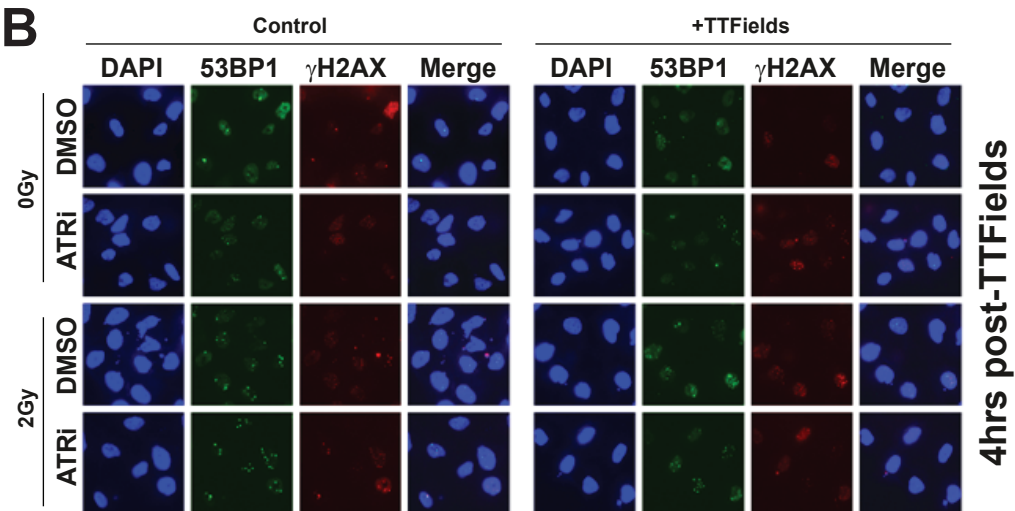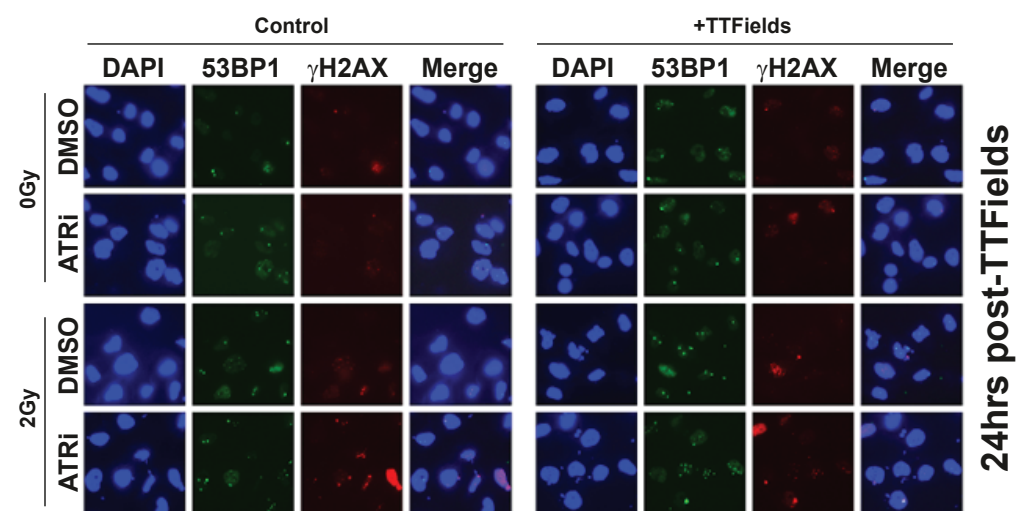

Supplement: Supplementary file 4 — Supplementary Figure S3 [file 41416_2023_2454_MOESM4_ESM.pdf]
